# Plasma protein profiling reveals candidate biomarkers for multiple sclerosis treatment

**DOI:** 10.1371/journal.pone.0217208

**Published:** 2019-05-29

**Authors:** Sahl Khalid Bedri, Ola B. Nilsson, Katharina Fink, Anna Månberg, Carl Hamsten, Burcu Ayoglu, Ali Manouchehrinia, Peter Nilsson, Tomas Olsson, Jan Hillert, Hans Grönlund, Anna Glaser

**Affiliations:** 1 Department of Clinical Neuroscience and Centrum for Molecular Medicine at Karolinska, Institutet, Stockholm, Sweden; 2 TCER AB, c/o Advice Företagsassistans i Stockholm AB, Stockholm, Sweden; 3 Department of Neurology, Karolinska University Hospital, Stockholm, Sweden; 4 Affinity Proteomics, SciLifeLab, School of Engineering Sciences in Chemistry, Biotechnology and Health, KTH-Royal Institute of Technology, Stockholm, Sweden; 5 Immunology and Allergy unit, Department of Medicine, Karolinska Institutet, Stockholm, Sweden; San Raffaele Scientific Institue, ITALY

## Abstract

Multiple sclerosis (MS) treatment options have improved significantly over the past decades, but the consequences of MS can still be devastating and the needs for monitoring treatment surveillance are considerable. In the current study we used affinity proteomics technology to identify potential biomarkers which could ultimately be used to as facilitate treatment decisions. We profiled the intra-individual changes in the levels of 59 target proteins using an antibody suspension bead array in serial plasma samples from 44 MS patients during treatment with natalizumab followed by fingolimod. Nine proteins showed decreasing plasma levels during natalizumab treatment, with PEBP1 and RTN3 displaying the most significant changes. Protein levels remained stable during fingolimod treatment for both proteins. The decreasing PEBP1 levels during natalizumab treatment could be validated using ELISA and replicated in an independent cohort. These results support the use of this technology as a high throughput method of identifying potentially useful biomarkers of MS treatment.

## Background

Multiple sclerosis (MS), is a chronic demyelinating inflammatory disease of the central nervous system (CNS) with both genetic and environmental factors involved in its development [1]. MS is one of the most common cause of neurological disability in young adults after trauma [2]. The treatment options for MS patients have improved significantly in the past decade and a number of immune-modulatory drugs are now available [3]. There are ongoing studies to determine the most optimal treatment strategies for individual MS patients [4]. This development emphasizes the need for suitable biomarkers to assist in making and monitoring treatment decisions.

Advancement in proteomics technology is rapid and the utilization of these technologies in the medical field both in clinical practice and research is expanding. Specially, following the progress in DNA microarray technology in the past two decades protein microarrays have developed rapidly[5]. Protein microarrays can be planar microarrays, where the capturing reagents are spotted on a glass slide, or bead based arrays, where capturing reagents are bound to color coded microspheres[6]. The use of these protein microarrays has allowed large scale profiling of protein expression in small volumes of human body fluids. This facilitates the investigation of a panel of candidate biomarkers instead of single ones, which could be useful in studying complex diseases where many factors are involved in the development and progress of the disease.

The antibody suspension bead array technology has previously been used together with antibodies generated within the Human Protein Atlas project (HPA, www.proteinatlas.org) [7] to explore biomarkers both in non-neurological diseases such as muscular and renal disorders [8, 9] and neurological diseases including amyotrophic lateral sclerosis and MS using this bead based array[10, 11]. These studies have successfully applied the method for protein profiling in plasma samples as well as cerebrospinal fluid (CSF) samples from MS patients [11, 12]. For neurological diseases, CSF has been the preferable body fluid to be profiled rather than plasma or serum, due to its close proximity to the CNS, but as lumbar puncture, the procedure for obtaining CSF, is an invasive procedure with potential risks it is difficult to obtain CSF more than occasionally. Previous studies in MS were mainly focused on samples taken at a single time point as a cross-sectional study [11, 12].

In the current study, we used the antibody suspension bead array system as a method to screen for potential biomarkers for MS treatment. We applied this method in a longitudinal manner on serial plasma samples from MS patients undergoing treatment. Protein profiling of serial samples from the same patient is more sensitive for studying intra-individual changes over treatment periods than inter-individual changes, and this serves the purpose of tailored medicine avoiding the issue of different protein levels in different individuals. Plasma samples were obtained from patients who were on natalizumab treatment and then switched to fingolimod due to risk of developing progressive multifocal leukoencephalopathy (PML)[13]. The selection of the proteins to be profiled in these samples was based on the pathways of recently established MS risk associated genetic variants [14] as well as markers of inflammation, expression in the CNS and inclusion in other protein profiling studies of MS [11, 12]. Identified changes in protein levels in plasma samples from MS patients could point to relevance of such specific proteins to MS treatment effects.

## Materials and methods

### Patient cohorts and plasma samples

A total of 44 MS patients were included first in the study as a screening cohort. These patients had been on natalizumab treatment for an average of 33 months and responded well to treatment and were then switched to a daily dosage of 500 mg fingolimod for various reasons, most commonly for being positive for antibodies to JC-virus, which carries a risk of developing PML [13]. Before starting fingolimod treatment the patients had a washout period of an average 2.5 months. Serial plasma samples from these patients were available before and during both treatment periods, enabling the examination of changes of the protein levels over treatment time.

Patients included in the study are part of the Immunomodulation and MS Epidemiology (IMSE) cohorts I and II at Karolinska Institutet, Sweden [15, 16]. Their plasma samples were collected in a longitudinal manner, beginning at treatment start and subsequently at 6, 12 and 24 months. Hence, for both natalizumab and fingolimod treatments there are a maximum of 8 plasma samples per patient included in this study. Patients´ clinical data was obtained from the Swedish MS registry, and included information on disability progression in the form of the expanded disability status scale (EDSS) and multiple sclerosis severity score (MSSS). Information about the MS patients and the plasma samples from the screening cohort is available in Table 1. and for the replication cohort (57 patients) in Table 2.

**Table 1:** Summary of demographic and clinical characteristics of MS patients included in the screening cohort. Median (interquartile range).

Gender female (%) = 32 (65.3)	Natalizumab treatment				Fingolimod treatment			
	Baseline	6 month	12 month	24 month	Baseline	6 month	12 month	24 month
n	38	37	37	29	26	26	26	8
Age at baseline	27.0 (21.4,29.9)				28.6 (22.0,35.0)			
*DD at baseline	8.5 (4.0,12.0)				11.0 (8.0,12.8)			
Cell count. 10^9^/L	NA	NA	NA	NA	3.0 (2.4,3.6)	0.5 (0.3,0.7)	0.5 (0.4,0.6)	0.8 (0.5,1.1)
EDSS	2.5 (2.0,3.5)	2.5 (1.5,3.5)	2.0 (1.0,3.0)	2.5 (1.5,3.5)	2.5 (1.5,3.5)	2.5 (2.0,3.5)	2.5 (2.0,4.0)	2.5 (2.0,5.0)
MSSS	4.2 (2.7,6.0)	3.4 (1.8,5.2)	2.7 (1.1,5.0)	2.6 (1.1,5.6)	2.7 (1.7,4.8)	2.9 (2.0,4.8)	2.6 (1.9,5.2)	2.0 (1.7,6.0)

*Disease duration at baseline

**Table 2:** Summary of demographic and clinical characteristics of MS patients included in the replication cohort. Median (interquartile range).

Gender female (%) = 32 (56.1)	Natalizumab treatment		
	Baseline	12 month	24 month
n	57	57	45
Age at baseline	36.0 (30.0,45.0)		
*DD at baseline	7.4 (3.8, 11.6)		
EDSS	2.5 (1.5, 3.5)	2.0 (1.5, 3.5)	2.0 (1.5, 3.5)
MSSS	4.2 (2.1, 5.9)	3.7 (1.5, 4.6)	2.9 (1.5, 4.1)

*Disease duration at baseline

MS patients on dimethyl fumarate (DMF) treatment (n = 35) were also included in the study. Their plasma samples were collected before and after 12 month of treatment as part of the IMSE V cohort in different hospitals around Sweden [17]. Plasma samples from age and gender matched healthy controls (HCs) (n = 38) included in this study are part of the Epidemiologic Investigation of Multiple Sclerosis (EIMS) study [18].

### Antibody suspension bead array

A total of 90 antibodies available within the HPA project were used to analyze 59 target proteins (Table A in S1 File). The bead array protocol was performed as previously described with minor adjustments[19]. Briefly, plasma samples were diluted 1:10 using phosphate buffer saline and labeled with biotin. The antibodies were coupled to magnetic color-coded beads, each representing an antibody. The biotin labeled samples were diluted 1:16 in assay buffer and heat treated at 56°C for 30 minutes. A mixture of the beads was distributed into 384-well plate, labeled samples were added, the plate subsequently washed and streptavidin conjugated fluorophore added. The plate was analysed in a FlexMap3D instrument (Luminex corp.). For each bead identity, results were reported in median fluorescence intensity (MFI).

### Validation

Antibodies for Phosphatidylethanolamine-Binding Protein 1 (PEBP1) and Reticulon 3 (RTN3) were validated using ELISA. For the PEBP1 antibody (HPA008819, Atlas antibodies), we coated 96-well plates with either PEBP1 peptide (APrEST71549, Atlas antibodies) or whole PEBP1 recombinant protein (NBP1-30224, Novus biologicals). After overnight incubation at room temperature the HPA008819 was added as the primary antibody followed by alkaline phosphatase conjugated anti-rabbit IgG (111-055-045, Jackson ImmunoResearch) as a secondary antibody and optical density measured. Sandwich ELISA was used by coating with a polyclonal goat anti-human PEBP1 (VPA00067, Bio-Rad) and after an overnight incubation at room temperature, plasma from MS patients or different concentrations of PEBP1 recombinant protein were added, followed by HPA008819 as a detection antibody. Some measurements of PEBP1 in plasma samples from natalizumab treated patients were below the level of detection of the ELISA. They were assigned a concentration of 0.1 μg/ml which is four folds lower than the last standard point concentration (0.39 μg/ml) and the same factor used for the serial dilution of the standard. The PEBP1 sandwich ELISA inter-assay coefficient of variance (CV) was 5.4% and the intra-assay CV for plasma samples was 2.6%. For the RTN3 antibody (HPA015649, Atlas antibodies), wells were coated with two RTN3 protein fragments covering different parts of the protein (Figure A in S2 File) that were produced in our lab (materials and methods in S1 File); the amino acids sequence from 2–299 (RTN3_A) (Figure B in S2 File) and the amino acids sequence from 254–700 (RTN3_B). The RTN3_B amino acid sequence incorporates the PrEST antigen sequence (Figure C in S2 File) that was used for the generation of HPA015649 in the HPA project. HPA015649 was used as a primary antibody. We performed an inhibition ELISA by first incubating HPA015649 in plasma samples then adding the mix to wells coated with the RTN3 fragment RTN3_B. RTN3 antibody not incubated in plasma samples was added to the control wells. The RTN3 inhibition ELISA inter-assay CV was 21,8% and the intra-assay CV for plasma samples was 2.5%.

### Statistical analysis

A linear mixed effect model was used to follow changes in protein plasma levels over time. This model takes into consideration the random effects between different patients and the fixed effects within the same patient. We used this to test for changes in protein levels and for association between these changes and clinical outcomes and to adjust for age, sex and disease duration. This was done using the “LmerTest” package [20]. The MFI data was normally distributed by transforming the data using Tukey’s ladder of powers and then centered. Transformation of the data was done using the “rcompanion” package. P values < 0.001 were considered statistically significant and the Bonferroni procedure was used for multiple testing correction using the “multitest” package [21]. A cutoff in the coefficient generated from the linear mixed effect model of 0.25 was used to select the proteins with the most pronounced change. Pearson's correlation was used to test for the correlation between the levels obtained from the antibody suspension bead array and the ELISAs. To test for the difference in PEBP1 and RTN3 levels between HCs and MS patients the Welch two sample t-test was used. This comparison was done between samples that were ran in ELISA plates that also included a control sample. Only plates that included a control sample with a level within the interquartile range calculated for all the ELISA runs were included in the HCs and MS patients’ comparison. All statistical analysis and graphs were done using R software version 3.3.2.

## Results

In this study, we applied a multiplexed affinity proteomics approach to generate protein profiles in plasma samples from patients on MS treatment. The selected antibodies were all chosen based on the corresponding target proteins being previously identified as relevant to MS development in genetic and/or protein profiling studies (Table A in S1 File). These antibodies generated protein profiles in serial plasma samples from MS patients when treated with natalizumab followed by fingolimod. The patients generally responded well to both natalizumab and fingolimod as indicated by disability scores (Table 1).

### Plasma protein profiles during treatment

**Fig 1:**
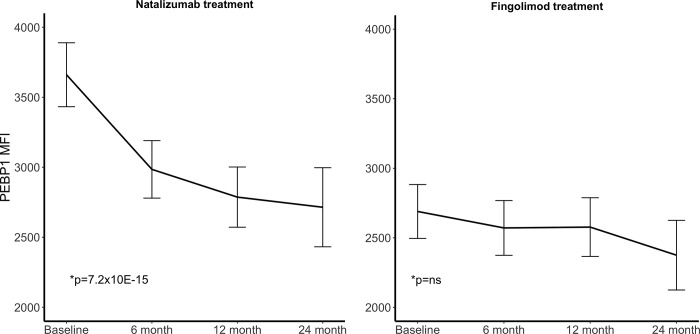
Changes in PEBP1 MFI levels during treatment. The line connects the mean of the MFI levels at each time point and the error bars indicate the standard error of the mean. *p value from the linear mixed model.

**Fig 2:**
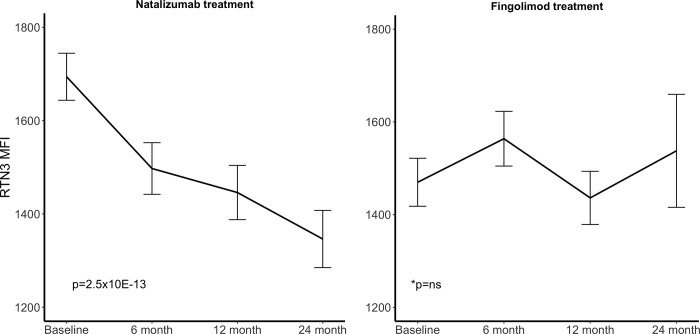
Changes in RTN3 MFI levels during treatment. The line connects the mean of the MFI levels at each time point and the error bars indicate the standard error of the mean. *p value from the linear mixed model.

To assess the impact of MS treatment on peripheral levels of selected proteins we used the linear mixed effect model to test for their changes during natalizumab and fingolimod treatment periods separately. Using cutoff for the coefficient of 0.25 and a p value < 0.001, revealed a total of 10 antibodies with significant changes in the levels of their target proteins during natalizumab treatment (Table 3). Two of the 10 significant antibodies target the protein RTN3 (HPA015649 and HPA015650). Noticeably, the levels of all nine proteins decreased during natalizumab treatment (Figure D in S2 File) and PEBP1 (HPA008819, p = 7,2x10^-15^) and RTN3 (HPA015649, p = 2,5x10^-13^) showed the most significant decline during natalizumab treatment (Figs 1 and 2). During fingolimod treatment none of the proteins showed any significant changes in plasma levels (Figs 1 and 2).

**Table 3:** The target proteins that changed significantly during natalizumab treatment.

Gene	Protein name	Antibody	Coefficient^a^	P value^b^	Proposed function
PEBP1	Phosphatidylethanolamine-Binding Protein 1	HPA008819	-0.26	7.2x10^-15^	-Precursor of hippocampal cholinergic neurostimulating peptide[22]. - Inhibits MAPK pathway and NF-κB pathway [23, 24].
RTN3	Reticulon 3	HPA015649	-0.30	2.5x10^-13^	Axonal development and autophagy[25]
AHI1	Abelson helper integration site 1	HPA057491	-0.26	1.4x10^-12^	Important for ciliary signaling during cerebellum development[26].
AFMID	-Arylformamidase -Kynurenine Formamidase	HPA061100	-0.30	1.8x10^-10^	Catalyzes the second step in the kynurenine pathway of tryptophan degradation[27].
SH3GL2	-SH3 Domain Containing GRB2 Like 2 -Endophilin A1	HPA063573	-0.26	7.7x10^-10^	Involved in neurotransmitter release and Clearance[28].
AMPH	Amphiphysin	HPA019829	-0.29	3.8x10^-8^	Involved in synaptic vesicles endocytosis[29].
RTN3	Reticulon 3	HPA015650	-0.27	2.9x10^-7^	Axonal development and autophagy[25]
IL12B	Interleukin 12B (p40)	HPA048230	-0.27	1.2x10^-6^	A subunit of IL12, it activates NK cells and promotes Th1 differentiation and INF-γ production[30].
GFAP	Glial fibrillary acidic protein	HPA056030	-0.25	4.9x10^-6^	Stability and morphology of astrocytes[31].
IL1F10	-Interleukin 1 family member 10 -Interleukin 38	HPA056887	-0.26	4.7x10^-5^	Anti-inflammatory and receptor antagonist for the IL-36R[32].

^a^ Coefficients are based on the transformed and centered data.

^b^ P values after correction for multiple testing using Bonferroni procedure.

### Validation of PEBP1 and RTN3 antibodies

In order to follow up on the most significant results from the initial protein profiling we wanted to validate antibody selectivity for PEBP1 and RTN3 respectively. For this purpose, we developed two in-house ELISAs for each antibody. We were able to show that HPA008819 binds both PEBP1 peptide and the recombinant protein (Figure E in S2 File) and that HPA008819 can detect PEBP1 in plasma samples from MS patients and recombinant protein in a concentration dependent manner (Figure E in S2 File). Similarly, we could show that HPA015649 was able to bind the RTN3 protein fragment RTN3_B (Figure F in S2 File) and that HPA015649 pre-incubation with plasma samples inhibited binding of the antibody to coated proteins in a manner consistent with the presence of RTN3 protein in plasma (Figure F in S2 File).

### Effects of disease modifying therapies on PEBP1 and RTN3 levels

**Fig 3:**
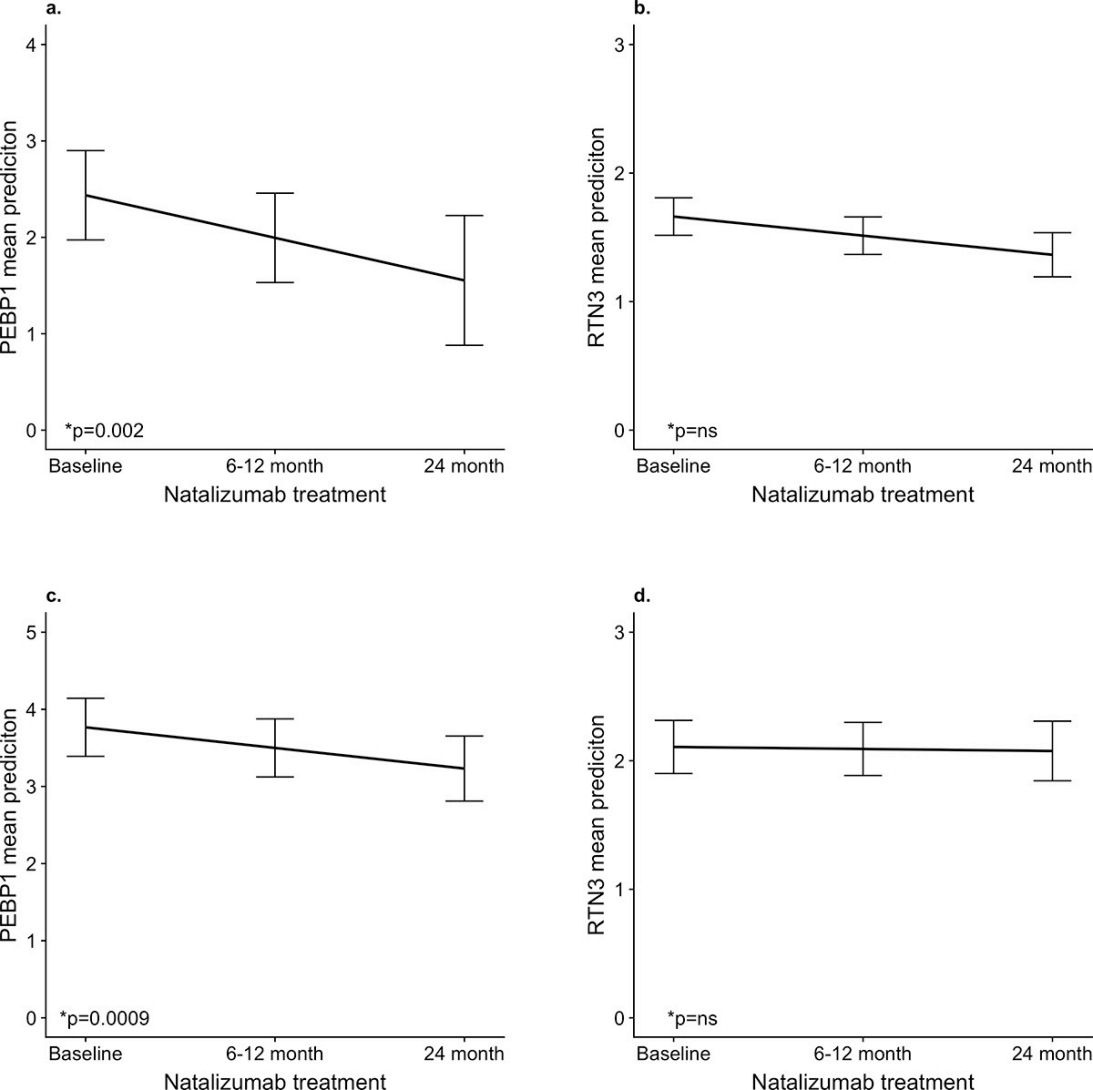
Validation of the changes in PEBP1 and RTN3 plasma levels during natalizumab treatment using ELISA. a) changes in PEBP1 in the screening cohort. b) changes in RTN3 in the screening cohort. c) changes in PEBP1 in the replication cohort. d) changes in RTN3 in the replication cohort. The Y axis represents the mean prediction of the change of PEBP1 and RTN3 levels during treatment obtained from the linear mixed model and the error bars indicate the standard error of the mean. *p value from the linear mixed model.

Using our in-house developed sandwich ELISA for PEBP1 and inhibition ELISA for RTN3, we measured the levels of PEBP1 and RTN3 in the plasma from 35 of the 44 patients that were included in the antibody suspension bead array analysis. The plasma samples were from natalizumab treatment start and at 6 or 12 and 24 months of natalizumab treatment. Applying the linear mixed effect model we detected a significant decrease in the levels of PEBP1 during natalizumab treatment (p = 0.004) (Fig 3A), validating the original observation for PEBP1 from the antibody suspension bead. We did not observe a significant decrease of RTN3 levels (Fig 3B). We tested for the correlation between the levels of PEBP1 and RTN3 obtained from the antibody suspension bead array and the ELISAs. There was a significant correlation between the two methods for PEBP1(p = 0.001, cor = 0.34) but not RTN3 (p = 0.477, cor = 0.08) (Figure G in S2 File). We speculate that the reason for not being able to reproduce the decline in RNT3 using ELISA was that the original the antibody suspension bead array results were based on heat treated samples which facilitates epitope accessibility. To test for this, we heat treated plasma samples at 56°C for half an hour and compared the RTN3 levels between heat treated and non-heat treated samples. Pairwise comparisons using Wilcoxon signed rank test showed that heat treating the samples does not significantly change the RTN3 levels (Figure H ins S2 File).

In an independent replication cohort of plasma samples from natalizumab treated MS patients (n = 57), we studied the changes in plasma PEBP1 and RTN3 during treatment. As in the screening cohort we observed a decrease in the levels of PEBP1 during treatment (p = 0.0009) (Fig 3C), while the levels of RTN3 did not change (Fig 3D).

**Fig 4:**
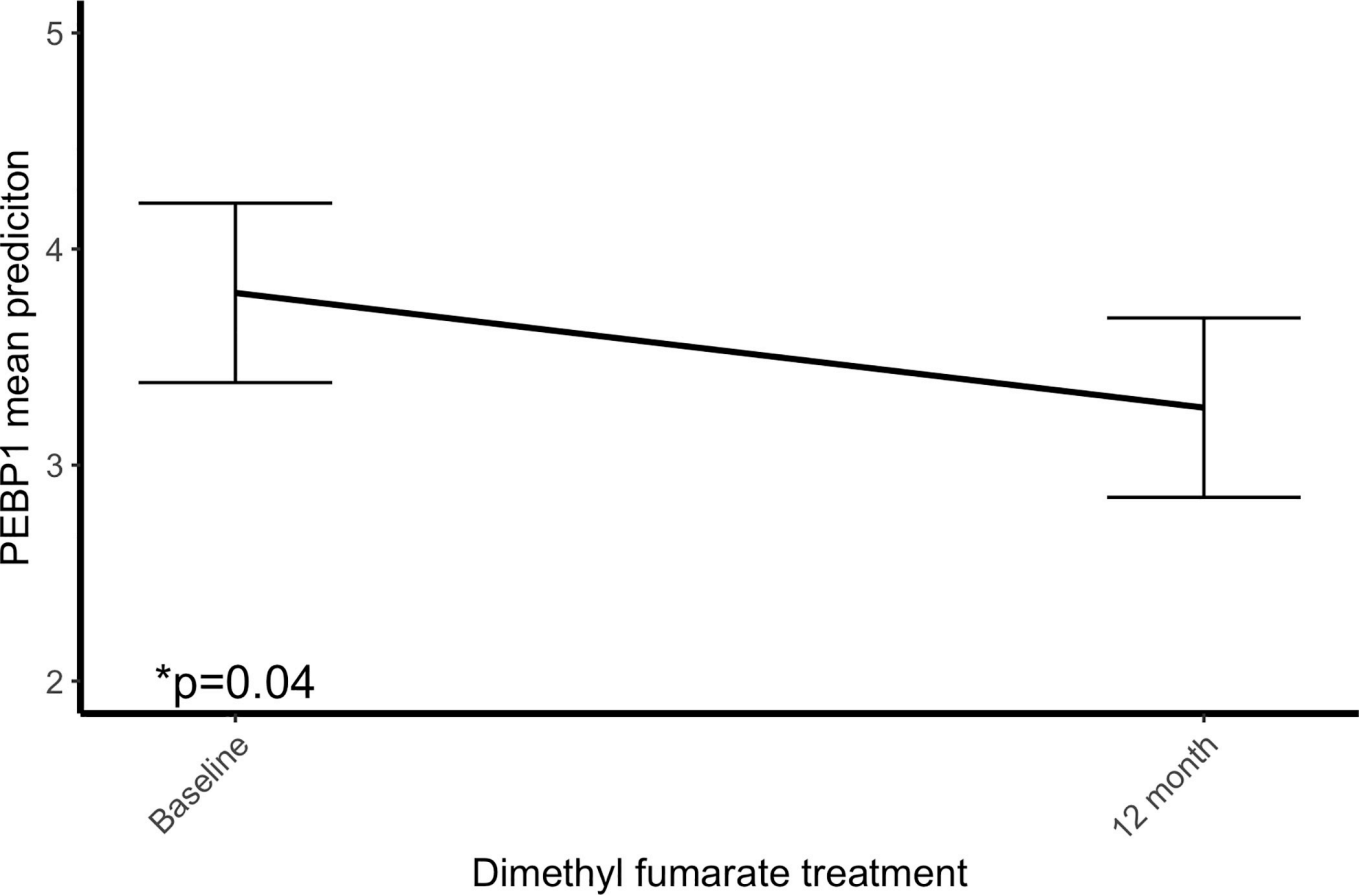
PEBP1 plasma levels before and after 12 month DMF treatment. The Y axis represents the mean prediction of the change of PEBP1 levels during treatment obtained from the linear mixed model and the error bars indicate the standard error of the mean. *p value from the linear mixed model.

After observing the decrease of PEBP1 during natalizumab treatment we were curious as to whether other immune-modulatory drugs will have the same effect on PEBP1. Therefore, in a group of DMF treated MS patients we measured the plasma levels of PEBP1 before and after 12 month of treatment. As in natalizumab treated patients the levels of PEBP1 decreased significantly after 12 month of treatment (p = 0.04) (Fig 4).

### Comparison of PEBP1 and RTN3 between HCs and MS patients

The plasma levels of PEBP1 and RTN3 were compared between HCs and MS patients at baseline and 6 or 12 month after natalizumab treatment initiation. We detected no difference in PEBP1 levels between the two groups before or during treatment (Table 4). RTN3 levels were significantly lower in MS patients than HCs at baseline and during treatment (Table 4).

**Table 4:** Comparison of PEBP1 and RTN3 between HCs and MS patients at baseline and 12 month natalizumab treatment. Mean (standard error of the mean).

	*PEBP1 μg/ml	^§^P value	*RTN3 μg/ml	^§^P value
HC	4.64 (0.25)		2.36 (018)	
Baseline	4.31 (0.41)	0.50	1.54 (0.15)	0.0006
12 month	3.94 (0.48)	0.21	1.39 (0.14)	0.00004

*Log transformed levels

^§^P value from the Welch Two Sample t-test.

### MS severity and PEBP1 and RTN3 plasma levels

**Fig 5:**
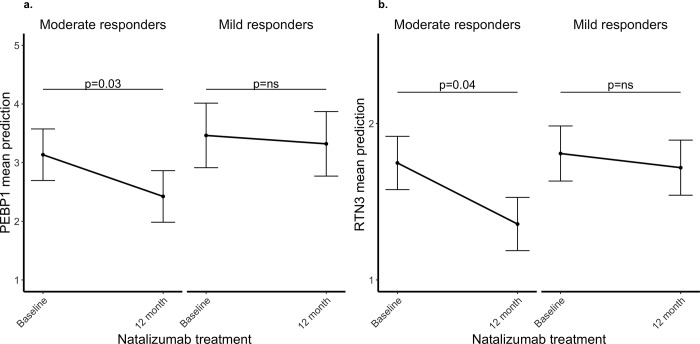
The changes in a) PEBP1 and b) RTN3 plasma levels and MS severity. Moderate responders are 20 patients with the highest decrease in MSSS during treatment. Mild responders are 20 patients with the lowest decrease in MSSS during treatment. The Y axis represents the mean prediction of the change of PEBP1 and RTN3 levels during treatment obtained from the linear mixed model and the error bars indicate the standard error of the mean. *p value from the linear mixed model.

By combining both the screening and replication cohort, we tested for an association of the clinical outcome MSSS and the change in the levels of PEBP1 and RTN3 after 12 month of natalizumab treatment. As the majority of MS patients respond to natalizumab treatment, to increase the sensitivity of the test, we tested for the association in 20 patients with the highest (moderate responders) and 20 patients with the lowest (mild responders) decrease in MSSS during treatment. The levels of both PEBP1 and RTN3 decreased significantly (p = 0.03, p = 0.04, respectively) in the moderate responders but did not change in the mild responders (Fig 5A and 5B). The decrease in PEBP1 and RTN3 was not significantly different between the moderate and mild responders.

## Discussion

In this study, the aim was to screen for biomarkers for MS treatment. For this purpose, we set out to explore protein profiles in serial plasma samples from MS patients during treatment periods of natalizumab followed by fingolimod. We could detect changes in the plasma levels for nine out of 59 targeted proteins during natalizumab treatment. For all these proteins the levels decreased over the treatment period and the proteins that displayed the most significant changes in plasma levels were PEBP1 and RTN3. The reliability of the results was further supported by the validation of the specificity of the antibodies towards PEBP1 and RTN3 using two different types of ELISA for each protein as well as reproducing the results of decreasing levels of PEBP1 plasma levels during natalizumab treatment. The decreasing levels of RTN3 during natalizumab treatment did not reach significance levels in the validation and we speculate that the reason could be related to ELISAs being performed on native proteins whilst the original HPA results were based on heat treatment of the samples which facilitates epitope accessibility[33]. However, when we compared heat treated and non-heat treated samples we did not detect any differences in RTN3 levels making this an unlikely explanation for the inability to reproduce the results using ELISA. Another possibility is that the inhibition ELISA was not specific enough for accurate measurement of RTN3 in plasma samples.

All the patients selected in the study responded well to natalizumab, and most of the patients responded well also to fingolimod. The decrease or lack of increase in EDSS and the decreasing MSSS values across the treatment period describes this favorable treatment responses (Table 1). With the current study design, we cannot tell if the decrease in plasma levels of PEBP1 during natalizumab is a treatment effect or a consequence of a reduced disability progression as a result of the treatment. However, we detect a significant decrease in PEBP1 and RTN3 levels in plasma from a group of patients with less disability progression during natalizumab treatment and this effect is not seen in another group of patients with more disability progression. We speculate that the changes in PEBP1 and RTN3 levels during treatment could be a reflection of disease modifying therapies. Once patients respond well to treatment these proteins may remain on a decreased level. Interestingly the decline in PEBP1 plasma was also detected in samples from MS patients on another highly efficient treatment, DMF.

To our knowledge, plasma levels of PEBP1 and RTN3 have not been previously studied and this could be the first study to have attempted to measure these levels in plasma Obtaining the normal range of plasma levels of these proteins might help addressing the question if their levels come down to a normal range after treatment. Intriguingly, we found the levels of RTN3 to be higher in HCs than in MS patients before and during natalizumab treatment, whilst for PEBP1, the levels were similar between HCs and MS patients before and during treatment. This might however be more of a reflection of the benefit of studying intra-individual changes in longitudinal cohorts rather than inter-individual changes in cross-sectional cohorts where general differences in plasma protein levels between people could obscure the results.

The peripheral measurement of PEBP1 in this study might be a reflection of the inflammation and degeneration in the CNS, especially that we see a reduction in the levels of PEBP1 after commencement of the highly efficacious second line immunomodulatory drugs. The reduction of PEBP1 levels after treatment might reflect the decrease of neurodegeneration as a consequence of the treatment.

PEBP1 has many functions, is expressed in many organs and is predominantly present in the hippocampus [34]. Acting as hippocampal cholinergic neurostimulating peptide (HCNP) precursor protein (HCNP-pp) from its N terminal the undecapeptide HCNP is derived which was reported to enhance acetylcholine synthesis in vitro by upregulating the production of choline acetyltransferase [22]. HCNP-pp expression was described to be lower in the hippocampus in Alzheimer's disease (AD) patients compare to non-demented patients [34] and its derivative HCNP was detected in the CSF of AD patients and reported to be high in some patients [35]. This highlights the importance of this protein for hippocampal memory activity. In the animal model of MS, MOG-induced experimental autoimmune encephalitis in Dark Agouti rats, PEBP1 was one of the down-regulated genes in the remitting stage of this disease model[36]. As RAF1 kinase inhibitor protein (RKIP) it inhibits the mitogen activated protein kinase (MAPK) pathway by binding and inhibiting RAF1 protein kinase and in addition it inhibits the NF-κB pathway, hence regulating these pathways of cell survival and anti-apoptotic activity [23, 24].

RTN3 belongs to the family of reticulon proteins that are involved in endoplasmic reticulum shaping and morphogenesis [25]. It is highly expressed in the brain [37], predominantly in neuronal bodies [38] and also in the axons and dendrites [39]. RTN3 is suggested to be involved in axonal development and autophagy[25]. RTN3 was observed to bind to and inhibit β-amyloid converting enzyme 1 (BACE1) [38]. Aggregates of RTN3 have been reported in the brain of AD patients [40] and other neurodegenerative diseases[41].

We explored the approach to test genes close to MS susceptibility loci as potential biomarkers, as we previously did for the IL7, whose expression was indeed found to be altered by recombinant interferon-beta treatment in MS[42]. Eleven of the targeted proteins in the present study were selected on the basis of their genes being identified as susceptibility loci for MS (Table A in S1 File). However, in this specific paradigm, from baseline, to natalizumab followed by fingolimod treatment, this group of gene products showed limited promise as biomarkers.

## Conclusions

In summary, we used a high-throughput screening proteomics method in order to identify biomarkers for MS treatment effects. The most significant findings from this initial analysis of plasma samples from MS patients revealed decreasing levels of several proteins during natalizumab treatment, where we were able to confirm the results for PEBP1. These results will need to be replicated in further studies, and suggest that this or similar methods could be used to screen for novel biomarkers in autoimmune diseases such as MS.

## Supporting information

S1 File Materials and methods. **Table A.** The selected target proteins gene IDs and the antibodies used for measuring them.
(DOCX)

S2 File **Figure A.** Schematic overview of the RTN3_A and RTN3_B constructs.
**Figure B.** RTN3_A protein sequence.
**Figure C.** RTN3_B protein sequence. The PrEST Antigen RTN3 is highlighted in yellow.
**Figure D.** Ten antibodies with significant changes in the levels of their target proteins levels during treatment.
**Figure E.** a) PEBP1 indirect ELISA results. b) PEBP1 sandwich ELISA results.
**Figure F.** a) RTN3 indirect ELISA results. b) RTN3 inhibition ELISA results.
**Figure G.** Correlation between measurements observed by the antibody suspension bead array analysis and sandwich ELISA.
**Figure H.** Effect of heat treating plasma samples at 56°C for half an hour on RTN3 detection.
(ZIP)

## Abbreviations

MS: Multiple sclerosis
CNS: central nervous system
CSF: cerebrospinal fluid
PML: progressive multifocal leukoencephalopathy
IMSE: Immunomodulation and MS Epidemiology
EDSS: expanded disability status scale
MSSS: multiple sclerosis severity score
PEBP1: Phosphatidylethanolamine-Binding Protein 1
RTN3: Reticulon 3
DMF: Dimethyl fumarate
