# Supplementary material for: Plasma protein profiling reveals candidate biomarkers for multiple sclerosis treatment
Source: PLoS One. 2019 May 29;14(5):e0217208. doi: 10.1371/journal.pone.0217208 (PMC6541274; doi:10.1371/journal.pone.0217208)
Supplement: S1 File — Table A. The selected target proteins gene IDs and the antibodies used for measuring them. (DOCX) [file pone.0217208.s001.docx]

| Table A. The selected target proteins gene IDs and the antibodies used for measuring them. The antibodies were available through the Human Protein Atlas project and they can be ordered from Atlas antibodies. | | | | | |
| --- | --- | --- | --- | --- | --- |
| Gene ID | **Antibody** | **Selection criteria** | **Gene** | **Antibody** | **Selection criteria** |
| *A1BG* | HPA044252 | Personal research interest | ***ITLN1*** | HPA063275 | Personal research interest |
| *A1BG* | HPA064407 | Personal research interest | ***MAG*** | HPA012499 | Brain expression |
| *ACPL2* | HPA017243 | Brain expression | ***MAP6*** | HPA039061 | Brain expression |
| *AFMID* | HPA026536 | Brain expression | ***MAP6*** | HPA039062 | Brain expression |
| *AFMID* | HPA061100 | Brain expression | ***MBP*** | HPA049222 | Brain expression |
| *AHI1* | HPA046684 | Brain expression | ***MBP*** | HPA064368 | Brain expression |
| *AHI1* | HPA057491 | Brain expression | ***MGAT5*** | HPA010919 | Previous studies[2] |
| *Albumin* | anti-Albumin1 | Personal research interest | ***MMEL1*** | HPA008205 | MS GWAS |
| *Albumin* | anti-Albumin2 | Personal research interest | ***MMP25*** | HPA055640 | Previous studies[7] |
| *AMPH* | HPA019828 | Brain expression | ***MOG*** | HPA021873 | Brain expression |
| *AMPH* | HPA019829 | Brain expression | ***NDFIP1*** | HPA009682 | MS GWAS |
| *ANO2* | HPA036276 | Previous studies[1] | ***NEFH*** | HPA061615 | Brain expression |
| *ANO2* | HPA036277 | Previous studies[1] | ***NEFM*** | HPA022845 | Brain expression |
| *ANO2* | HPA072555 | Previous studies[1] | ***NEFM*** | HPA023138 | Brain expression |
| *AQP4* | HPA014782 | Brain expression | ***OMG*** | HPA008206 | Brain expression |
| *AQP4* | HPA014784 | Brain expression | ***PEBP1*** | HPA008819 | Brain expression |
| *C1orf106* | HPA027499 | MS GWAS | ***PEBP1*** | HPA063904 | Brain expression |
| *C1orf106* | HPA027511 | MS GWAS | ***PLG*** | HPA048823 | Personal research interest |
| *C3* | HPA003563 | Inflammatory marker | ***PLGLB1*** | HPA053770 | Personal research interest |
| *C3* | HPA020432 | Inflammatory marker | ***POMC*** | HPA063644 | Brain expression |
| *C3a* | AF3677 | Inflammatory marker | ***PROK2*** | HPA041408 | Personal research interest |
| *CD14* | HPA002035 | Inflammatory marker | ***PTPRZ1*** | HPA015103 | Brain expression |
| *CD14* | HPA002127 | Inflammatory marker | ***RTN3*** | HPA015649 | Brain expression |
| *CLEC16A* | HPA035815 | MS GWAS | ***RTN3*** | HPA015650 | Brain expression |
| *CLEC16A* | HPA061385 | MS GWAS | ***S100B*** | HPA015768 | Brain expression |
| *CNP* | HPA023278 | Brain expression | ***SDK2*** | HPA014725 | Personal research interest |
| *CNP* | HPA023280 | CNS | ***SERPINA1*** | HPA000927 | Previous studies[5] |
| *CNP* | HPA023338 | CNS | ***SERPINA3*** | HPA000893 | Previous studies[5] |
| *DKKL1* | HPA047194 | MS GWAS | ***SH3GL2*** | HPA026685 | Brain expression |
| *DKKL1* | HPA064797 | MS GWAS | ***SH3GL2*** | HPA063573 | Brain expression |
| *CSF3* | HPA001412 | Inflammatory marker | ***TAGAP*** | HPA031000 | MS GWAS |
| *GAP43* | HPA013392 | Previous studies[3, 5] | ***TAGAP*** | HPA039982 | MS GWAS |
| *GFAP* | HPA056030 | Brain expression | ***TIMMDC1*** | HPA053214 | MS GWAS |
| *GFAP* | HPA063513 | Brain expression | ***TIMMDC1*** | HPA055846 | MS GWAS |
| *ICAM5* | HPA008943 | Brain expression | ***TNFRSF6B*** | HPA047154 | Personal research interest |
| *ICAM5* | HPA009083 | Brain expression | ***TNFSF14*** | HPA012700 | MS GWAS |
| *IFI30* | HPA026650 | MS GWAS | ***TRIM2*** | HPA035853 | Brain expression |
| *IgG* | Anti-IgG | Inflammatory marker | ***TRIM2*** | HPA035854 | Brain expression |
| *IL12A* | HPA001886 | MS GWAS | ***UNC80*** | HPA042472 | Brain expression |
| *IL12B* | HPA048230 | Inflammatory marker | ***UNC80*** | HPA043612 | Brain expression |
| *IL1F10* | HPA056887 | Inflammatory marker | ***UNC80*** | HPA050959 | Brain expression |
| *IL1F10* | HPA059813 | Inflammatory marker | ***WBSCR17*** | HPA013624 | Brain expression |
| *IL2RA* | HPA054622 | MS GWAS | ***WBSCR17*** | HPA047986 | Brain expression |
| *IL6* | HPA060030 | Inflammatory marker | ***ZPBP2*** | HPA027997 | Personal research interest |
| *IRF8* | AF5117 | Previous studies[3] | ***ZPBP2*** | HPA053070 | Personal research interest |

# **Materials and methods**

## Design and cloning of RTN3 constructs

Recombinant RTN3 (UniProtKB - O95197) was constructed by dividing the first 700 amino acids (aa) of the protein coding sequence in two parts (see Figure S3 for details), designated RTN3_A (Figure S4) and RTN3_B (Figure S5), and purchasing each part as a recombinant gene (Thermo Fisher Scientific). The signal peptide was removed from the open reading frame of RTN3_A using SignalP (<http://www.cbs.dtu.dk/services/SignalP/>). The fragments were subcloned into a modified pBad expression vector (Thermo Fisher Scientific) using golden gate cloning as described[4], resulting in fusion protein containing a 6x HIS-tag for purification and a 2x Albumin binding domain (2xABD035[6]) for expression levels and solubility.

## Production and purification of RTN3_A and RTN3_B

The vector DNA containing the RTN3_A and RTN3_B constructs were transformed into *E. coli* BL21-AI cells (Thermo Fisher Scientific). Cells were grown in 500 ml Super broth medium supplemented with 100 mg/L Ampicillin, 1 mM Mg_2_SO_4_, 0.6% Glycerol, 0.2% Arabinose and 0.015% Glucose for auto induction at 25°C over night. The cells were spun down (10000g), and pellets were dissolved in 2.5ml lysis buffer (6 M Guanidinium-HCl, 10mM Tris-HCl, 50mM NaHPO_4_, 100 mM NaCl and 20 mM betamercaptoethanol with pH set to 8.0) per gram pellet. Cells were frozen in -80°C until completely frozen and thawed shaking at 37°C until completely thawed. The lysates were centrifuged at 23500 g for 60 min and pellets were discared while supernatants were stored at 8°C in new tubes until purification.

1.8 ml of each protein supernatant fraction was loaded to one tube with His Mag Sepharose® Ni Beads (GE Healthcare) previously equilibrated with lysis buffer and incubated end-over-end for >60 minutes. The supernatants were discarded and the beads were washed twice with 1.5ml wash buffer A (6M Urea, 500mM NaCl, 0.05% Tween 20, 10 mM betamercaptoethanol, 10 mM MES, pH 6.7), and once with 1.5ml wash buffer B (6M Urea, 0.05% Tween 20, 10 mM betamercaptoethanol, 10 mM MES, pH 6.7). Finally, the proteins were eluted three times with 300 µl elution buffer (6M Urea, 0.05% Tween 20, 10 mM betamercaptoethanol, 10 mM MES). The eluated fractions were immediately pH adjusted to pH5 by adding a pre-calibrated amount (8.6% of the elution volume) of 0.82M MES buffer to each fraction. The eluted fractions were analysed for purity using SDS-PAGE and concentration of the eluted proteins was estimated using NanoDrop (Thermo Fisher Scientific).

# **References**

[1] B. Ayoglu, N. Mitsios, I. Kockum, M. Khademi, A. Zandian, R. Sjöberg, B. Forsström, J. Bredenberg, I. Lima Bomfim, E. Holmgren, H. Grönlund, A.O. Guerreiro-Cacais, N. Abdelmagid, M. Uhlén, T. Waterboer, L. Alfredsson, J. Mulder, J.M. Schwenk, T. Olsson, P. Nilsson, Anoctamin 2 identified as an autoimmune target in multiple sclerosis, Proc Natl Acad Sci U S A 113 (2016) 2188-2193.

[2] B. Brynedal, J. Wojcik, F. Esposito, V. Debailleul, J. Yaouanq, F. Martinelli-Boneschi, G. Edan, G. Comi, J. Hillert, H. Abderrahim, MGAT5 alters the severity of multiple sclerosis, J Neuroimmunol 220 (2010) 120-124.

[3] S. Bystrom, B. Ayoglu, A. Haggmark, N. Mitsios, M.G. Hong, K. Drobin, B. Forsstrom, C. Fredolini, M. Khademi, S. Amor, M. Uhlen, T. Olsson, J. Mulder, P. Nilsson, J.M. Schwenk, Affinity proteomic profiling of plasma, cerebrospinal fluid, and brain tissue within multiple sclerosis, J Proteome Res 13 (2014) 4607-4619.

[4] C. Engler, R. Kandzia, S. Marillonnet, A one pot, one step, precision cloning method with high throughput capability, PLoS One 3 (2008) e3647.

[5] A. Haggmark, S. Bystrom, B. Ayoglu, U. Qundos, M. Uhlen, M. Khademi, T. Olsson, J.M. Schwenk, P. Nilsson, Antibody-based profiling of cerebrospinal fluid within multiple sclerosis, Proteomics 13 (2013) 2256-2267.

[6] A. Jonsson, J. Dogan, N. Herne, L. Abrahmsén, P.A. Nygren, Engineering of a femtomolar affinity binding protein to human serum albumin, Protein Eng Des Sel 21 (2008) 515-527.

[7] S.A. Shiryaev, A.G. Remacle, A.Y. Savinov, A.V. Chernov, P. Cieplak, I.A. Radichev, R. Williams, T.N. Shiryaeva, K. Gawlik, T.I. Postnova, B.I. Ratnikov, A.M. Eroshkin, K. Motamedchaboki, J.W. Smith, A.Y. Strongin, Inflammatory proprotein convertase-matrix metalloproteinase proteolytic pathway in antigen-presenting cells as a step to autoimmune multiple sclerosis, J Biol Chem 284 (2009) 30615-30626.
