# Supplementary material for: Plasma protein profiling reveals candidate biomarkers for multiple sclerosis treatment
Source: PLoS One. 2019 May 29;14(5):e0217208. doi: 10.1371/journal.pone.0217208 (PMC6541274; doi:10.1371/journal.pone.0217208)
Supplement: S2 File — Figure A. Schematic overview of the RTN3_A and RTN3_B constructs. Figure B. RTN3_A protein sequence. Figure C. RTN3_B protein sequence. The PrEST Antigen RTN3 is highlighted in yellow. Figure D. Ten antibodies with significant changes in the levels of their target proteins levels during treatment. Figure E. a) PEBP1 indirect ELISA results. b) PEBP1 sandwich ELISA results. Figure F. a) RTN3 indirect ELISA results. b) RTN3 inhibition ELISA results. Figure G. Correlation between measurements observed by the antibody suspension bead array analysis and sandwich ELISA. Figure H. Effect of heat treating plasma samples at 56°C for half an hour on RTN3 detection. (ZIP) [file pone.0217208.s002.zip › S2.docx]

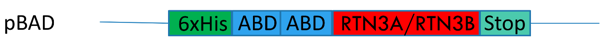


Fig A. Schematic overview of the RTN3_A and RTN3_B constructs

MKAIFVLKGSHHHHHHGSAQHDEAEAKVLANRELDKYGVSDFYKRLINKAKTVEGVEALKLHILAALPGGSGGSEAKVLANRELDKYGVSDFYKRLINKAKTVEGVEALKLHILAALPSGSGGSAEPSAATQSHSISSSSFGAEPSAPGGGGSPGACPALGTKSCSSSCADSFVSSSSSQPVSLFSTSQEGLSSLCSDEPSSEIMTSSFLSSSEIHNTGLTILHGEKSHVLGSQPILAKEGKDHLDLLDMKKMEKPQGTSNNVSDSSVSLAAGVHCDRPSIPASFPEHPAFLSKKIGQVEEQIDKETKNPNGVSSREAKTALDADDRFTLLTAQKPPTEYSKVEGIYTYSLSPSKVSGDDVIEKDSPESPFEVIIDKAAFDKEFKDSYKESTDDFGSWSVHTDKESSEDISETNDKLFPLRNKGGS-stop

Fig B. RTN3_A protein sequence

MKAIFVLKGSHHHHHHGSAQHDEAEAKVLANRELDKYGVSDFYKRLINKAKTVEGVEALKLHILAALPGGSGGSEAKVLANRELDKYGVSDFYKRLINKAKTVEGVEALKLHILAALPSGSGGSKAAFDKEFKDSYKESTDDFGSWSVHTDKESSEDISETNDKLFPLRNKEAGRYPMSALLSRQFSHTNAALEEVSRCVNDMHNFTNEILTWDLVPQVKQQTDKSSDCITKTTGLDMSEYNSEIPVVNLKTSTHQKTPVCSIDGSTPITKSTGDWAEASLQQENAITGKPVPDSLNSTKEFSIKGVQGNMQKQDDTLAELPGSPPEKCDSLGSGVATVKVVLPDDHLKDEMDWQSSALGEITEADSSGESDDTVIEDITADTSFENNKIQAEKPVSIPSAVVKTGEREIKEIPSCEREEKTSKNFEELVSDSELHQDQPDILGRSPASEAACSKVPDTNVSLEDVSEVAPEKPITTENPKLPSTVSPNVFNETEFSLNVTTSAYLESLHGKNVKHIDDSSPEDLIAAFTETRDKGIVDSERNAFKAISEKMTDFKTTPPVEVLHENESGG-stop

Fig C. RTN3_B protein sequence. The PrEST Antigen RTN3 is highlighted in yellow.


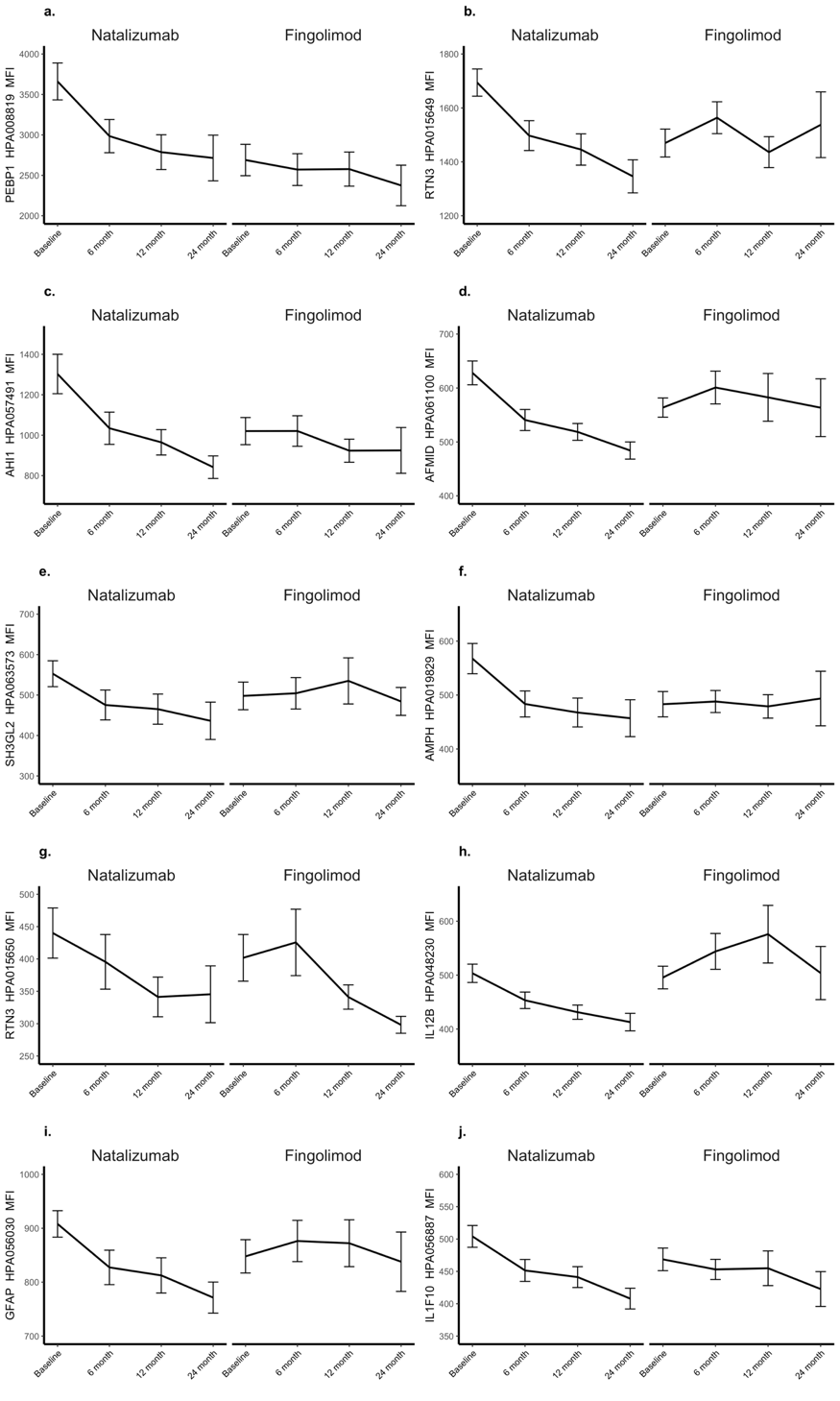


Fig D. Ten antibodies with significant changes in the levels of their target proteins levels during treatment. a) PEBP1 HPA008819, b) RTN3 HPA015649, c) AHI1 HPA057491, d) AFMID HPA061100, e) SH3GL2 HPA063573, f) AMPH HPA019829, g) RTN3.HPA015650, h) IL12B HPA048230, i) GFAP HPA056030, j) IL1F10 HPA056887. The line connects the mean of the MFI levels at each time point and the error bars indicate the standard error of the mean.


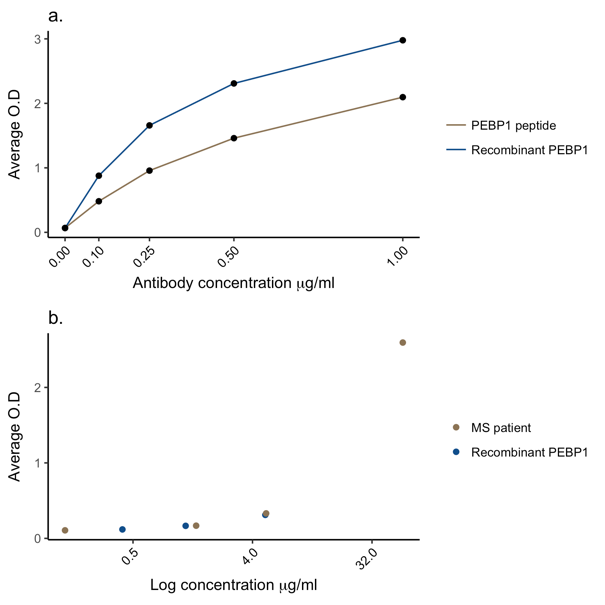


Fig E. a) PEBP1 indirect ELISA results. 96 well plate was coated with PEBP1 peptide or recombinant PEBP1 and HPA008819 was used as a primary antibody. Optical density measurements were taken using a filter of 405nm. b) PEBP1 sandwich ELISA results. Recombinant PEBP1 in serial dilutions or plasma from MS patients were added to 96 wells plate coated with polyclonal goat anti-human PEBP1 (VPA00067, Bio-Rad). HPA008819 was used as a detection antibody. Optical density measurements were taken using a filter of 405nm.


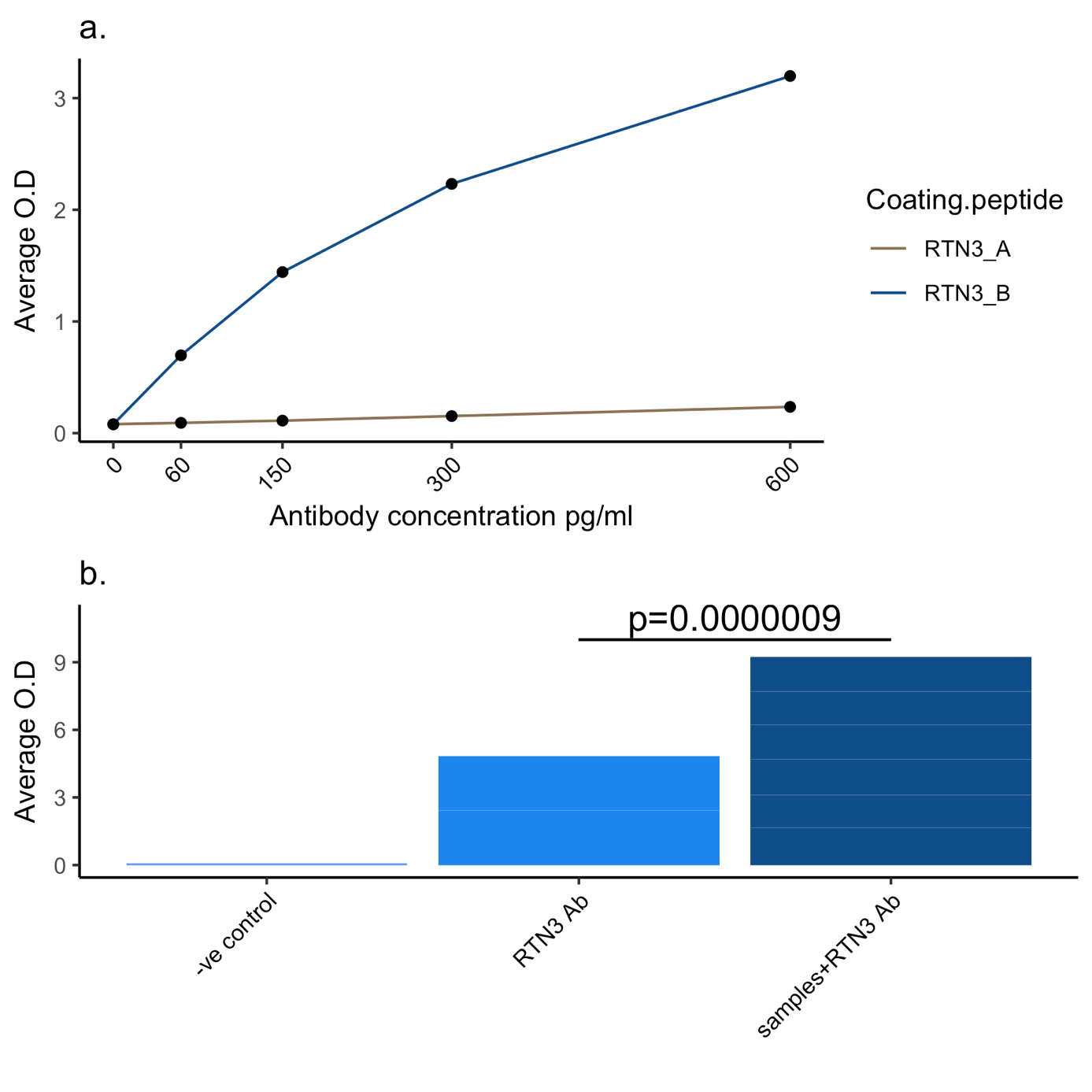


Fig F. a) RTN3 indirect ELISA results. 96 well plate was coated with RTN3 peptides; RTN3_A peptide or RTN3_B. and HPA015649 was used as a primary antibody. Optical density measurements were taken using a filter of 405nm. b) RTN3 inhibition ELISA results. HPA015649 incubated in plasma samples (samples+RTN3 Ab) or only a plasma sample (-ve control) or only HPA015649 (RTN3 Ab) were added to 96 wells plate coated with the peptide RTN3_B. Optical density measurements were taken using a filter of 405nm.

P value from the Welch Two Sample t-test


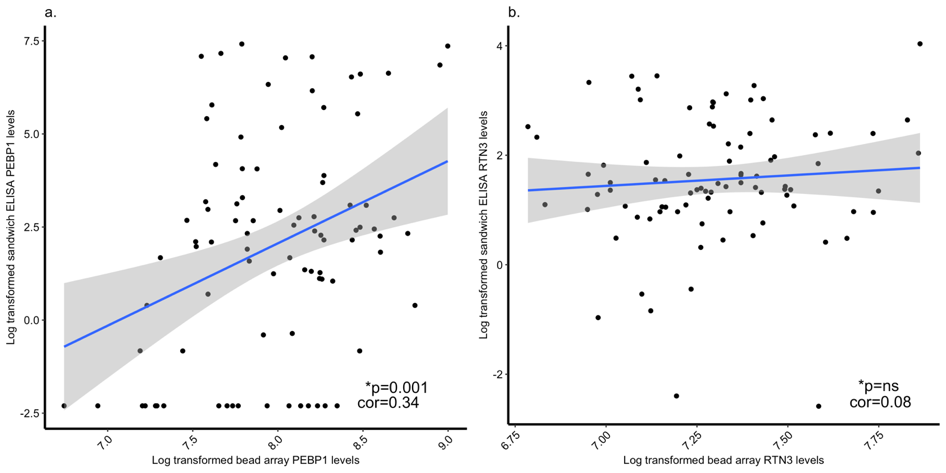


Fig G. Correlation between measurements observed by the antibody suspension bead array analysis and sandwich ELISA.

*p value from Pearson's correlation.


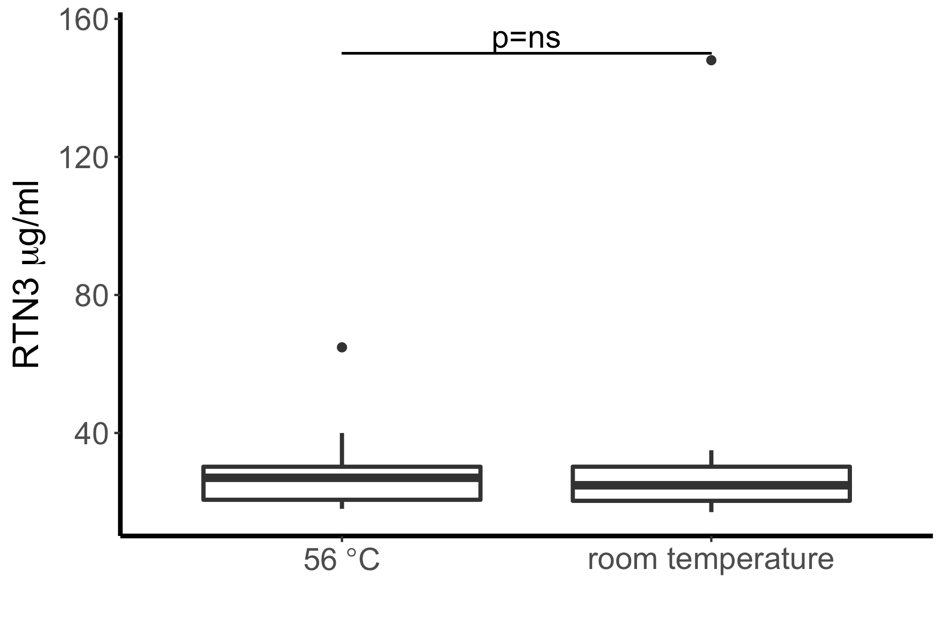


Fig H. Effect of heat treating plasma samples at 56ºC for half an hour on RTN3 detection.

p value from Pairwise comparisons using Wilcoxon signed rank test.
